# Spatial distribution of decadal ice-thickness change and glacier stored water loss in the Upper Ganga basin, India during 2000–2014

**DOI:** 10.1038/s41598-019-53055-y

**Published:** 2019-11-13

**Authors:** Debmita Bandyopadhyay, Gulab Singh, Anil V. Kulkarni

**Affiliations:** 1 Center of Studies in Resources Engineering, Indian Institute of Technology Bombay, Mumbai, India; 2 Divecha Centre for Climate Change, Indian Institute of Science, Bangalore, India

**Keywords:** Cryospheric science, Environmental impact

## Abstract

Himalayan glaciers have long been the focus of glaciologists across the world while trying to understand the contrasting patterns of elevation and mass changes. However, with limited number of ground observations, a comprehensive assessment of mass balance on a regional scale still remains elusive. Using the synoptic coverage of remote sensing data, we estimate a detailed spatial variation of glacier ice thickness change in the Central Himalaya of Uttarakhand using geodetic method, on a catchment scale. High resolution TerraSAR-X/TanDEM-X (12 m) and SRTM (30 m) digital elevation models (DEMs) have been utilized. The mean elevation change in the catchments is found to be −9.56 ± 0.2 m (mean annual elevation change rate is −0.68 ± 0.01 m a^−1^). To highlight the water potential of this region, the total ice mass loss has been estimated to be 16.0 ± 1.2 Gigatonne (Gt) from 2000–2014 from eight identified catchments namely Yamunotri, Bhagirathi, Mandakini, Alaknanda, Dhauliganga, Pindar, Goriganga and Kali/Sarda. The estimated mass balance has been validated using reported observations on five selective glaciers and the coefficient of determination is 0.93. This spatial variation of ice thickness estimated in the eight catchments is critical, as the melt-water from these glaciers contribute to the upper Ganga basin.

## Introduction

The Himalaya span from the Karakoram to the Eastern Himalaya, over 2000 km^1^, making it one of the largest glacier mountain systems of the world. The Indian Himalayan glaciers have not only been of socio-economic importance, in terms of providing freshwater downstream, hydropower or acting as a climatic barrier but, have also been held responsible for devastating calamities like floods and mudslides owing to its continuous deglaciation. Such response of the Himalayan glaciers could be attributed to the accelerated rate of climate change. In fact, glaciers are identified as one of the most sensitive indicators of climate change^1^, hence an ideal subject when such studies are considered. In an agrarian economy like India, apart from rainwater, people are hugely dependent on freshwater which comes from the basins of Ganges, Indus and Brahmaputra. In other words, the Indian Himalayan glaciers play a vital role in controlling the economy of the country^2–4^. Therefore, it is imperative that glacier health in terms of thickness and volume changes be monitored to understand the amount of glacial wastage in a quantitative manner.

Meltwater resulting from glacier mass changes account for most of the hydrological process, from which the water storage capacity of the Himalayan glaciers could also be estimated^5^. However, for Himalaya, detailed large-scale quantification is yet to be performed. Such studies are important as there is a need to understand the current freshwater reserve of the major basins of the country. The basin characteristics of the glaciers are attributed to its size, elevation and mass change. Mass change studies incorporate change in area, elevation, alongside ice/snow/firn density consideration, which makes it one of the most sought after indicators of glacier health. Conventional methods like Ground Penetrating Radar (GPR), stake height observations or hydrological records, are the most direct methods of measuring the change in mass balance. Yet, these observations are sparsely distributed leading to more uncertainty when considering the entire glacier extent. For better coverage, high resolution satellite imagery (both optical and Synthetic Aperture Radar (SAR)) and techniques such as gravimetry, altimetry or interferometry SAR (InSAR) have been utilized^6^. Geodetic method helps in estimation of elevation changes using DEMs, which can be directly utilized for mass change predictions as well. Previous geodetic studies in the Indian Himalaya have been accomplished using various remote sensing satellites like ICESat laser altimetry^1,7^, SRTM^7–9^, ASTER^10,11^, Cartosat stereo pairs^9,12^, SPOT data^7,8,13–15^ and recently (since 2010) TanDEM-X/TerraSAR-X DEMs^16–18^. The major advantage of the TanDEM-X/TerraSAR-X pair is that the dataset provides the first space-borne interferometric SAR pair with a continuous single-baseline InSAR data which can be harnessed to generate accurate DEMs. Moreover, this dataset has a vertical accuracy of ~2 m which supersedes any other available DEM^22^.

**Figure 1:**
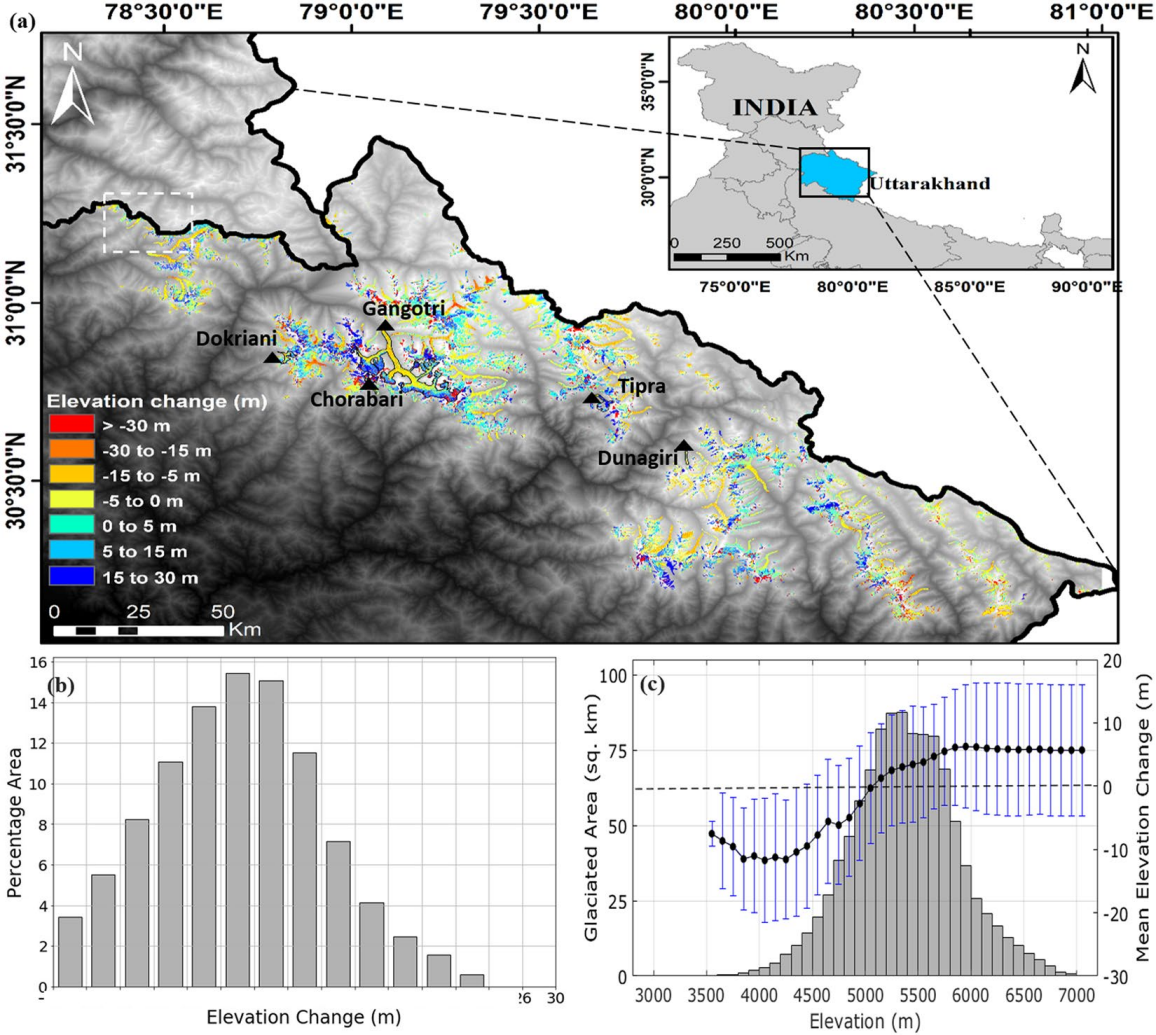
(**a**) Elevation change for glaciers in Uttarakhand (Central Himalaya) with the white dashed rectangle highlighting the area used for estimation of SRTM-X and C-band penetration difference. (**b**) histogram of the overall glacier elevation change in entire Uttarakhand region (**c**) hypsometry for all the glaciers of Uttarakhand (The maps were made using ArcGis 10.1 and the plots made using Matlab 2018a).

In the present study we evaluate the potential of SRTM and TanDEM-X/TerraSAR-X DEM products for monitoring Central Himalayan glaciers in India. A detailed spatial distribution of the elevation changes over the decade, 2000–2014 has been estimated over the entire state of Uttarakhand using high resolution DEMs. The glaciers in Uttarakhand cover mostly the Central Himalaya which stretches from 28°42′N to 31°28′N and 77°35′E to 81°05′E (Fig. 1a). The region has over 2000 glaciers feeding water into the major catchments of the state namely Yamunotri, Upper Bhagirathi, Upper Alaknanda, Mandakini, Dhauliganga, Pindar, Goriganga and Upper Kali/Sarda covering a total area of approximately 12000 km^2^. These glaciers are mainly fed by the summer monsoon precipitation and winter snow, with maximum precipitation from December to March, mostly due to the western disturbances^19^. The annual average rainfall recorded in the last five years has been 1431 mm^20^. For our study, five glaciers have been selected for validation of mass change results, and to understand the effect of glacier size on glacier elevation change. Lastly, to account for the glacial wastage and water potential, catchment-wise mass-budget has also been calculated. Such study on a catchment scale in Uttarakhand has not been reported yet and hence this information will certainly strengthen our understanding of the glaciers in this region.

## Dataset

The SRTM operated a 10-day mission in 2000 providing global DEMs using simultaneously operating X-band and C-band systems. The SRTM mission was jointly carried out by the US National Aeronautics and Space Administration (NASA) and the German Space Agency (DLR), in partnership with the US National Imagery and Mapping Agency (NIMA). The C-band dataset were processed by JPL (Jet Propulsion Laboratory) while the X-band processed and distributed at DLR^21^. Due to limited coverage of the SRTM X- band data over the glaciated region of Uttarakhand, elevation change studies have been carried using SRTM-C and TanDEM-X DEM. However, the penetration bias of C- and X- band have been considered using SRTM C- and X- band data for glaciated and non-glaciated regions.

TanDEM-X DEMs acquired in 2014 have been provided by DLR over the Uttarakhand range of Himalayan glaciers. The TanDEM-X mission with its twin satellite flying in helical-formation records amplitude and phase information with negligible time lag due to which there is near-zero temporal decorrelation^22^. Furthermore, penetration of X-band is hardly 40 cm considering the wetness (0.5% by vol.) of the snowpack covered glacier area for different seasons^23,24^. Hence, TanDEM-X data, would give a clearer picture of the surface properties of the glacier that are changing with time and hence utilized in this study. The glacier outlines have been used from the Randolph Glacier Inventory (RGI 6.0)^25^ database, which was created between 2001 and 2011(updated continuously). Since the study period is from 2000–2014, with the base year being 2000, the glacier outlines are manually corrected wherever necessary using Landsat imagery. The extent to which the boundary had to be corrected is shown in Supplementary Fig. S1.

Since, the elevation changes are related to the temperature fluctuation in the observation period, 2000–2014, Landsat thermal band (Landsat 5 and 7 for year 2000 and Landsat 8 for the year 2014) was utilized. To ascertain our analysis, we even used the high resolution ERA5 reanalysis product for the time period 2001–2014 (data is available since 2001) which is kindly provided by Copernicus Climate Change Service.

## Methodology

### DEM generation and bias correction

The prime step in geodetic method requires two-time period DEMs. For the year 2000, 30 m SRTM DEM has been used and for the year 2014, high resolution (12 m) TanDEM-X DEMs (TDM DEM) provided by DLR have been utilized. However, for Bhagirathi catchment, 12 m TDM DEM was unavailable. Hence, TerraSAR-X/TanDEM-X CoSSC products have been utilized to generate DEMs of 12 m resolution for this catchment only (Bhagirathi). To maintain planimetric consistency, all the DEMs were co-registered at same spatial reference system of WGS 84/UTM zone 44 with same spatial resolution (details are in Supplementary Section 1). This process facilitates removal of horizontal and vertical offset in the two DEMs that are being compared. For the regions where DEMs have been generated, the accuracy assessment is performed using the methodology used by Deo *et al*.^26^. As mentioned earlier, for penetration bias correction, SRTM-C and SRTM-X DEMs were used for glaciated and non-glaciated terrain (details in Supplementary Section 1).

### Mass budget calculation

Upon implementation of bias correction, the decadal elevation changes from 2000–2014 were obtained by DEM differencing. Modified RGI 6.0 glacier outlines were used to extract elevation change on glaciers and this was further converted into volume change (using glacier area). Accuracy assessment of the elevation change results for each catchment as well as the uncertainty owing to the manual delineation of the glacier boundaries are performed and discussed in Supplementary Section 3. Finally, the mean volume change (for each catchment) is converted into mass budget using a constant density conversion factor of 850 ± 60 kg m^−3^ for ice/firn for the entire Central Himalayan glaciers of Uttarakhand.

## Results and Discussion

### Mean elevation change

#### Elevation change of Uttarakhand glaciers

Quantification of surface elevation change for the Central Himalayan glaciers was done for the decade 2000–2014 (Fig. 1a). It was observed that the mean elevation change rate for the ~1950 glaciers is −0.68 ± 0.01 m a^−1^. The mean elevation change of glaciers in Uttarakhand as seen from the graph (Fig. 1b) show that the elevation change is more negatively skewed which accounts for more ice-mass loss in the glaciers. Further, the percentage of glaciated area where more ice-mass loss occurs is also higher. However the overall elevation change in the accumulation zone is positive (Fig. 1c), implying that the glaciers in this region are fairly sustainable in the near future.

The total mean elevation changes of the eight catchments of Uttarakhand (Central Himalaya) is −9.56 ± 0.2 m from 2000–2014 with an elevation change rate of −0.68 ± 0.01 m a^−1^. It is observed that ~15.45% (as seen in Fig. 1b) of total glaciated area has an elevation change of −7.93 ± 0.2 m from 2000–2014 where the mean glacier area is more than 9.54 km^2^. For uncertainty estimate, the spatial autocorrelation distance using global statistics has been estimated to be 1480 m, which facilitates the Normalized Absolute Mean Deviation (NMAD) calculation (detailed consequences of this estimate are provided in Supplementary Section 3).

#### Mean elevation change of eight catchments in Uttarakhand

**Figure 2:**
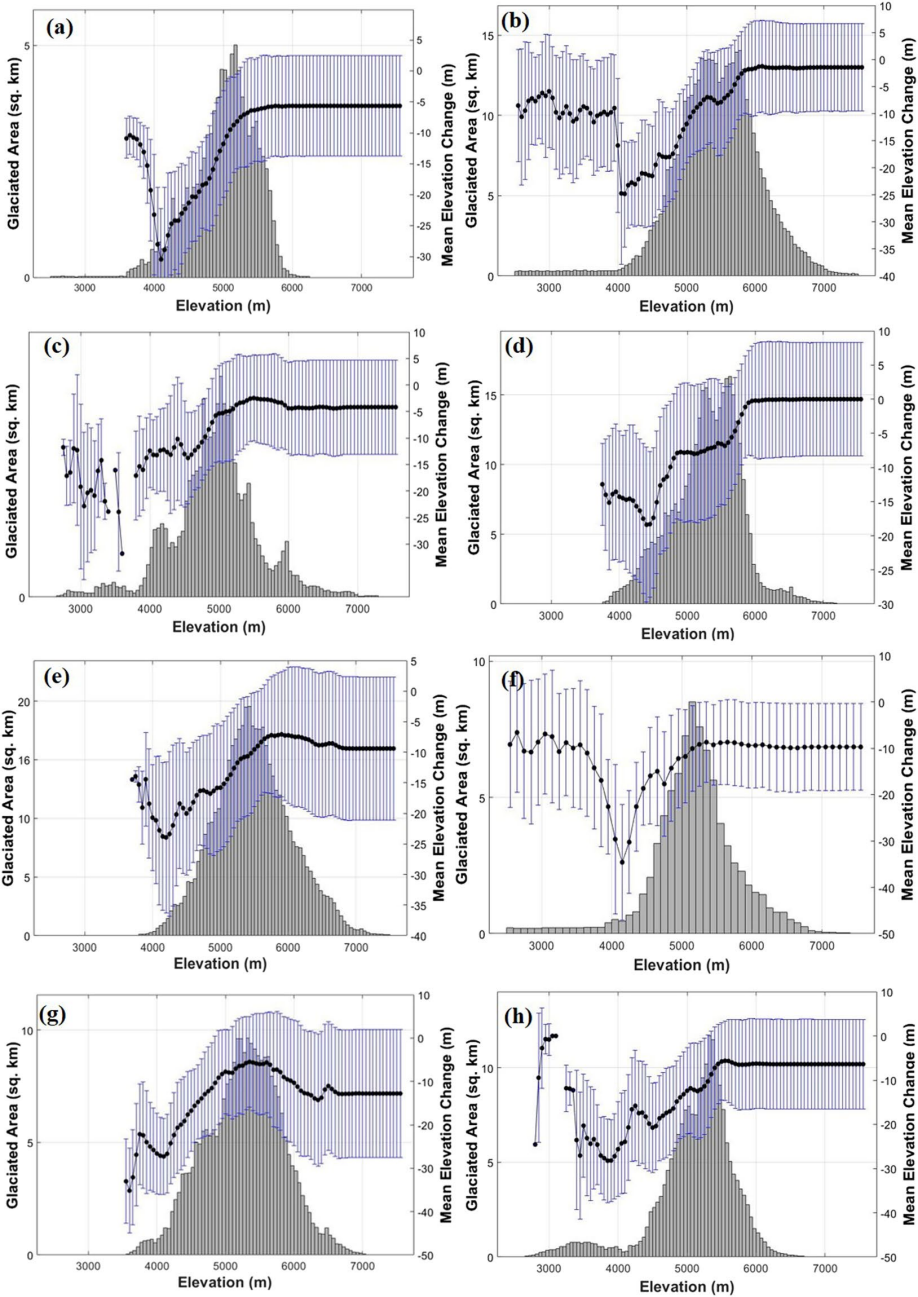
Altitudinal distribution of mean elevation change for the eight catchments (**a**) Yamunotri (**b**) Upper Bhagirathi (**c**) Mandakini (**d**) Upper Alaknanda (**e**) Dhauligana (**f**) Pindar (**g**) Goriganga (**h**) Upper Kali/Sarda plotted against glaciated area in the primary Y-axis (The plots were made using Matlab R2018a).

**Figure 3:**
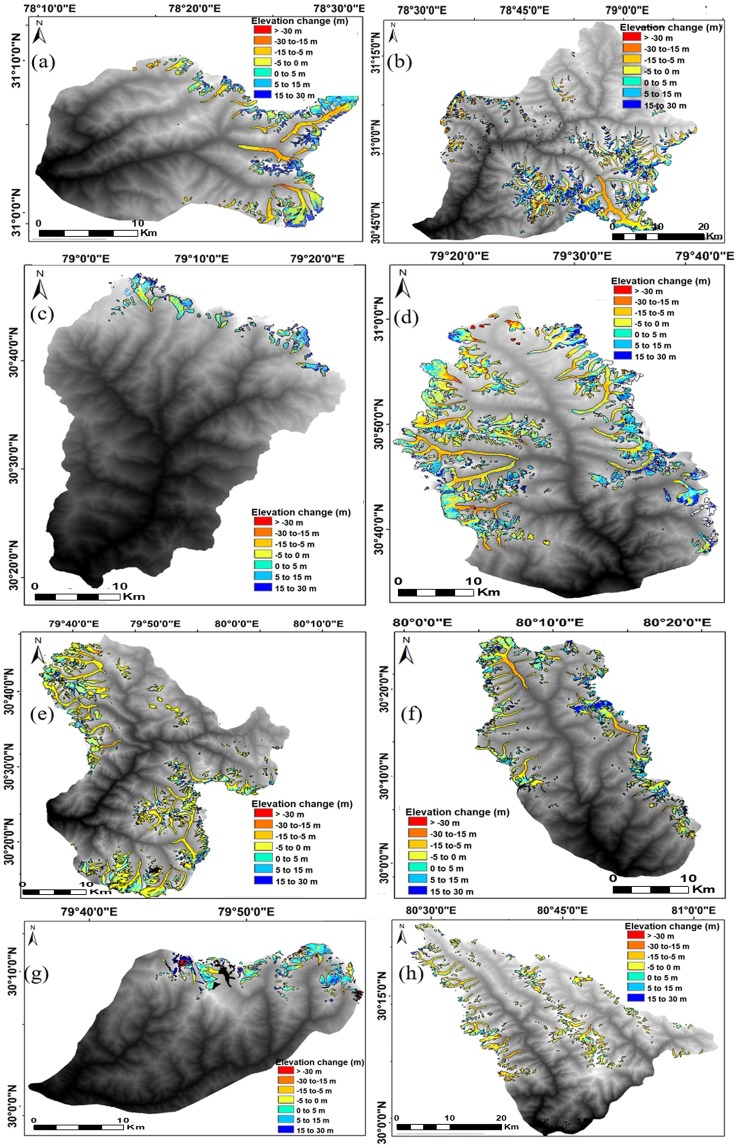
Elevation change in all eight catchments from 2000 to 2014 (**a**)Yamunotri (**b**) Upper Bhagirathi (**c**) Mandakini (**d**) Upper Alaknanda (**e**) Dhauliganga (**f**) Goriganga (**g**) Pindar and (**h**) Upper Kali/Sarda catchment. With elevation changes varying from −30 m (in red) to 30 m (in dark blue) (The maps were made using ArcGis10.1).

The altitudinal distribution of the elevation change in each of the catchments has been shown in Fig. 2. The elevation bins are made at every 100 m and the plot shows as to how the elevation changes (secondary Y-axis) for every 100 m from ~3000–7000 m. Also, the area of the glacier covered at each bin is shown in the hypsometry plots (primary Y-axis). Overall elevation change in all the eight catchments from 2000–2014 is shown in Fig. 3. As seen in Fig. 2, Bhagirathi holds the maximum catchment area and glaciated area but the mean elevation change is much lower with respect to other catchments. Further, the elevation changes are generally lower beyond 5000 m, which indicates that the Equilibrium Line Altitude (ELA) is near that altitude. The ablation zone shows contrasting patterns of ice-thickness change with altitude, which leads us to investigate the nature of local meteorological factors in that region. This heterogeneous nature is supported by the Accumulation Area Ratio (AAR) calculated for all the catchments (Supplementary Table S2). The AAR is indicative of the change in mass balance and ELA. As the AAR reduces, ELA shifts up, to a higher altitude, reducing the accumulation area of the glacier. Except Alaknanda, all other catchments have a decrease in AAR from 2000 to 2014 forcing the glaciers towards a negative mass balance. Alaknanda has an increase in AAR, which is further supported by the lowest mean temperature compared to all other catchments. However, elevation changes in the ablation zone are higher, leading to an overall loss in glacier thickness in Alaknanda. The AAR in the eight catchments varies from 0.11(in Pindar) to 0.56 (in Yamunotri). In fact, such a low AAR in Pindar is one of the major reasons for the highest elevation change in this catchment.

Maximum elevation change is seen in Mandakini and Pindar catchment (Supplementary Fig. S2), however, the glaciated area in these two catchments is less than any other catchment. Hence the contribution of Mandakini and Pindar towards the mass budget is significantly low (as seen in Table 1). Conversely, elevation change is maximum towards eastward region i.e. in Upper Kali/Sarda catchment (Fig. 3h). Possible reason for this elevation change pattern could be the increased influence of nearby monsoon-arid transition zone^7^. Since, the mean elevation changes for the glaciers of Central Himalaya (India) have not been reported, except for a few selective glaciers, hence validation of our results has been restricted only to these selective glaciers (Supplementary Fig. S1j–n). The elevation changes have been observed to follow a typical behavior of higher elevation change in the ablation zone and lower in the accumulation zone. This can be attributed to higher air temperature at lower elevations in addition to lower albedo of bare ice in the ablation zone. There might be only a few glaciers which have a reduced melt in the ablation zone and higher elevation change in the higher altitudes. This could be explained by the presence of thick debris cover in the ablation zone which acts as an insulator to the incoming solar radiation. A prominent example of this type of glacier is the Chorabari glacier (Supplementary Fig. S1k) wherein, the elevation change in most parts of the ablation zone is moderately lower. With a debris cover of >1 m, this glacier in fact shows no terminus retreat during the study period 2003–2010^27^. However, over multi-decade (1976–2016) the glacier has shown significant retreat rate^18^. Hence, the debris cover thickness can either reduce or enhance the glacier ice melt. However, this behavior is restricted to individual glaciers as large-scale information about debris cover is unavailable. On the contrary, there are certain catchments like the Yamnotri, Upper Alaknanda, Dhauliganga and Goriganga which shows a general trend in spatial distribution of elevation change across ablation and accumulation zones (i.e. higher elevation changes in the ablation zone and lower at higher altitudes) which cannot be explained by the theory of varying debris cover alone.

**Table 1:** Details of Catchment wise elevation change and mass budget along with glaciated area during 2000–2014.

Catchment	Glaciated area (km^2^)	Mean Elevation Change (m)	Mass Budget (Gt)
Yamunotri	107.13	−11.87 ± 0.85	−1.08 ± 0.07
Upper Bhagirathi	791.16	−5.76 ± 0.85	−3.87 ± 0.27
Mandakini	37.40	−4.82 ± 0.91	−0.15 ± 0.01
Upper Alaknanda	403.82	−5.23 ± 0.89	−1.80 ± 0.12
Dhauliganga	492.96	−12.02 ± 1.05	−5.04 ± 0.36
Pindar	75.97	−14.95 ± 1.05	−0.97 ± 0.06
Goriganga	247.49	−9.30 ± 1.22	−1.96 ± 0.14
Upper Kali/Sarda	198.03	−12.52 ± 1.12	−2.11 ± 0.15

To further investigate a more generic cause for this heterogeneous elevation change pattern, the surface temperature for entire Uttarakhand region (encompassing all the eight catchments) was analyzed. This was performed using the thermal band of Landsat (Supplementary Fig. S3) for 2000–2014 as well as the climate reanalysis product (ERA5) (please refer Supplementary Table S3) for the period, 2001–2014 (data of ERA5 is available since 2001). Except Alaknanda catchment, it was observed that the mean temperature in all the catchments increased from 2000 to 2014. This directly impacts the magnitude of the glacier elevation change as seen in Table 1. The mean temperature was calculated for September (i.e. summer ablation month) for both 2000 and 2014. Nevertheless, Pindar and Mandakini already have a higher mean temperature (1.75 °C and 1.01 °C respectively in 2014) which leads to more glacier ice-melt, thereby explaining the high glacier elevation change in these two regions as compared to other catchments.

#### Mean elevation change for selective glaciers of Uttarakhand

For validation, five glaciers with varying size (Gangotri (141 km^2^), Chorabari, Tipra and Dokriani (~7 km^2^ each) and Dunagiri (2.5 km^2^)) were chosen. The mean elevation change for these glaciers are shown in Supplementary Fig. S4j–n. The mean elevation changes for large glaciers (e.g. most part of Gangotri glacier area) falls in the range of 0 to −15 m, on the other hand the medium sized glaciers (Chorabari, Dokriani and Dunagiri) have a variable range of elevation change with maximum region falling under 5 to −5 m category. Certain regions (mostly near the end of ablation zone) show a mean elevation change of −5 to −15 m. In addition, small glaciers (<3 km^2^) such as the Dunagiri glacier have maximum region with a higher elevation range i.e. −5 to −15 m. This indicates that small sized glaciers have a higher effect on the glacier melt which is supported by previous studies^28,29^.

#### Geodetic mass balance for 2000–2014

**Figure 4:**
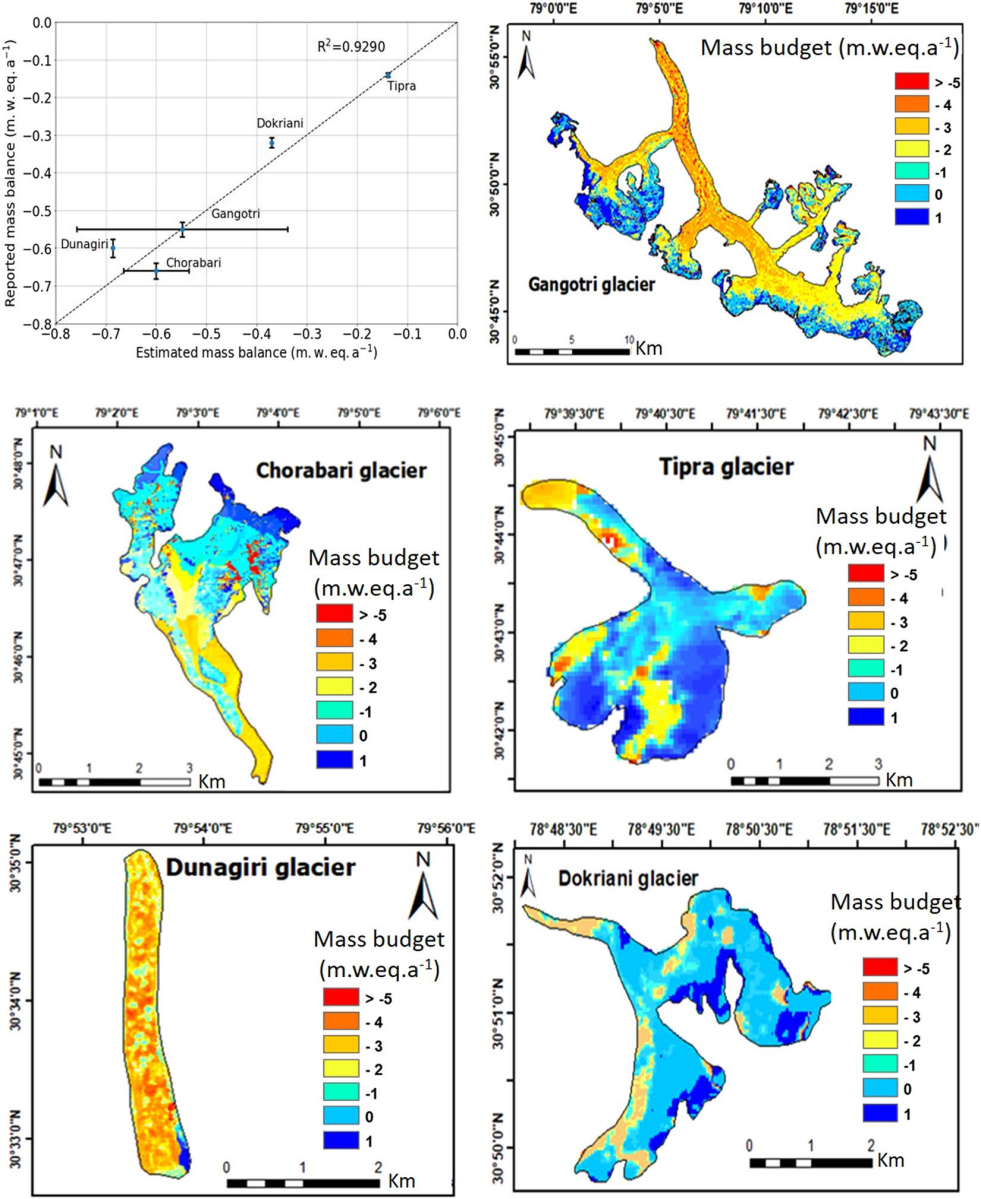
Comparison of mass balance with published measurements for the five selective glaciers namely Gangotri, Chorabari, Tipra, Dunagiri, Dokriani glacier in Uttarakhand estimated for 2000–2014 time period. (The plot was made using Python 3.7 and the maps made using ArcGis 10.1).

Mass budget for the entire Central Himalayan glaciers of India (the Upper Ganga basin,Uttarakhand) for the time period 2000–2014 has been found to be −1.21 ± 0.11 Gt a^−1^ (details of catchment-wise calculations are reported in Table 1). Of the five selected glaciers, geodetic mass balances have been reported for Gangotri and Chorabari glacier in the similar time frame as the period of study, hence are crucial for the purpose of validation. Dokriani glacier, Tipra glacier and Dunagiri glacier even though report glaciological mass balances of a historical time frame compared to current study period, it facilitates the understanding of the pattern of the glacier elevation changes and consequently the changes in mass balance. Figure 4 shows the comparison of mass balance estimates with the published measurements. The coefficient of determination is observed to be 0.93 and the error range in our estimates is below than the reported results.

While Bhushan *et al*.^9^ estimated the specific mass balance of Gangotri as −0.55 ± 0.42 m. w. eq. a^−1^ from 2006–2014, our results project a similar specific mass change of −0.54 ± 0.03 m. w. eq. a^−1^ for the period 2000–2014. Further, in our previous study^18^, a specific mass balance of −0.66 ± 0.13 m. w. eq. a^−1^ for the decade 2000–2016 was reported for Chorabari glacier, whereas here we estimate for 2000–2014 the specific mass balance as −0.63 ± 0.04 m. w. eq. a^−1^. As both the glaciers have comparable estimates as reported in previous studies, it validates our results. Specific mass balance of other glaciers like Dokriani^30^, Tipra^31^ and Dunagiri^32^ show that our estimates are within the error limits of that have been reported in earlier studies (Fig. 4). We, therefore, carry forward this methodology for further analysis in catchment-wise study.

For the mass budget estimated in this region, there are no observations reported in this time period, apart from the mass budget for Dhauliganga basin. Satter *et al*.^33^ report a mass budget of −3.3 ± 0.5 Gt considering 15 glaciers under the region of study whereas, our mass balance estimates (−5.04 ± 0.36 Gt) considering the entire glaciated terrain of Dhauliganga. Moreover, Kääb *et al*.^1^, published a mass budget of −4.7 Gt a^−1^ for 2003–2009 for Uttarakhand, Himachal Pradesh and West Nepal region. Our study assimilates the contribution from all the catchments in Uttarakhand as −1.21 ± 0.11 Gt a^−1^, providing an additional information of the contribution of glaciers, particularly in this state. However, the mass budget estimated by Kääb *et al*.^1^ used the sparse ICESat data coverage which are generally interpolated over large regions. In fact, Kääb *et al*.^1^ itself suggests that the mass change could be higher than the actual value. Moreover, the penetration bias calculated between ICESat and SRTM assumes negligible changes in elevation between 2000–2003 and 2003–2009, which is a big offset considering glacier dynamics. Thus, a disparity in results might seem plausible.

Seasonal as well as glacier-melt runoff have been considered parallel contributors to the water availability downstream. However, the latter acts as a reservoir of water locked up in the form of ice/snow. The water received in the form of precipitation is subject to the seasonal changes but the glacier assimilates solid-water annually on a multi-decadal scale. Hence, if the mass loss of the glaciers accelerates, the availability of water on a long term would be severely affected. With this point of concern, this study estimates the mass budget in terms of Gt of mass loss per year on a catchment scale that enables us to comprehend the extent of sustainable water in the near future. Besides, the meltwater from the glaciers of this region contribute approximately 12% to the Upper Ganga basin^34^ which further highlights the importance of such detailed catchment-wise study.

## Conclusion

In this study, we present a detailed spatial variation of glacier elevation and mass changes on a decadal scale in the Upper Ganga basin, India. Further, a catchment-wise mass budgeting was done to account for contribution of these glaciers to various tributaries of the Ganga basin on a regional scale. The mass budget for the eight catchments namely Yamunotri, Upper Bhagirathi, Mandakini, Upper Alaknanda, Dhauliganga, Pindar, Goriganga and Upper Kali/Sarda has been calculated, which range from −0.15 ± 0.01 Gt to −5.04 ± 0.36 Gt. The mean weighted mass balance is calculated to be −0.61 ± 0.04 m.w.eq. a^−1^ which is equivalent to 16.0 ± 1.2 Gt of glacier stored water loss from the Upper Ganga basin during 2000–2014. This information has significant relevance for various glacio-hydrological studies in future. In fact, this study is suggested to be integrated with the current database wherein only valley stations (point data) or gridded data are utilized, for inferring the relationship between mass balance and precipitation. This amalgamation shall certainly help in eliminating the error caused by under-estimation owing to limited number of data points representing the entire region of study as in the case of using ICESat data. The elevation change observations presented in this study for the Central Himalayan glaciers in Uttarakhand have not been documented before. Hence, this paper provides an important share of information to the existing knowledge of mass change studies over Indian Himalaya.

## Supplementary information

Spatial distribution of decadal ice-thickness change and glacier stored water loss in the Upper Ganga basin, India during 2000-2014

## Data Availability

The dataset utilized/analyzed during the current study will be available from corresponding author upon request.
