# Author Correction: Spatial distribution of decadal ice-thickness change and glacier stored water loss in the Upper Ganga basin, India during 2000–2014

**DOI:** 10.1038/s41598-019-55881-6

**Published:** 2019-12-12

**Authors:** Debmita Bandyopadhyay, Gulab Singh, Anil V. Kulkarni

**Affiliations:** 1 Center of Studies in Resources Engineering, Indian Institute of Technology Bombay, Mumbai, India; 2 Divecha Centre for Climate Change, Indian Institute of Science, Bangalore, India

Correction to: *Scientific Report* 10.1038/s41598-019-53055-y, published online 13 November 2019

At one point in the Article, the Authors incorrectly cite the wrong paper by Bhushan and colleagues in the ‘Geodetic mass balance for 2000–2014’ subsection of the Results and Discussion. The correct paper is included below as ref. 1. As a result,

“While Bhushan *et al*.^9^ estimated the specific mass balance of Gangotri as −0.55 ± 0.42 m. w. eq. a^−1^ from 2006–2014, our results project a similar specific mass change of −0.54 ± 0.03 m. w. eq. a^−1^ for the period 2000–2014.”

should read:

“While Bhushan *et al*.^1^ estimated the specific mass balance of Gangotri as −0.55 ± 0.42 m. w. eq. a^−1^ from 2006–2014, our results project a similar specific mass change of −0.54 ± 0.03 m. w. eq. a^−1^ for the period 2000–2014.”

All other citations to Reference 9 in the Article are correct.

In addition, in Figure 1b, the values on the X-axis are missing. The correct Figure 1 appears below.

**Figure 1:**
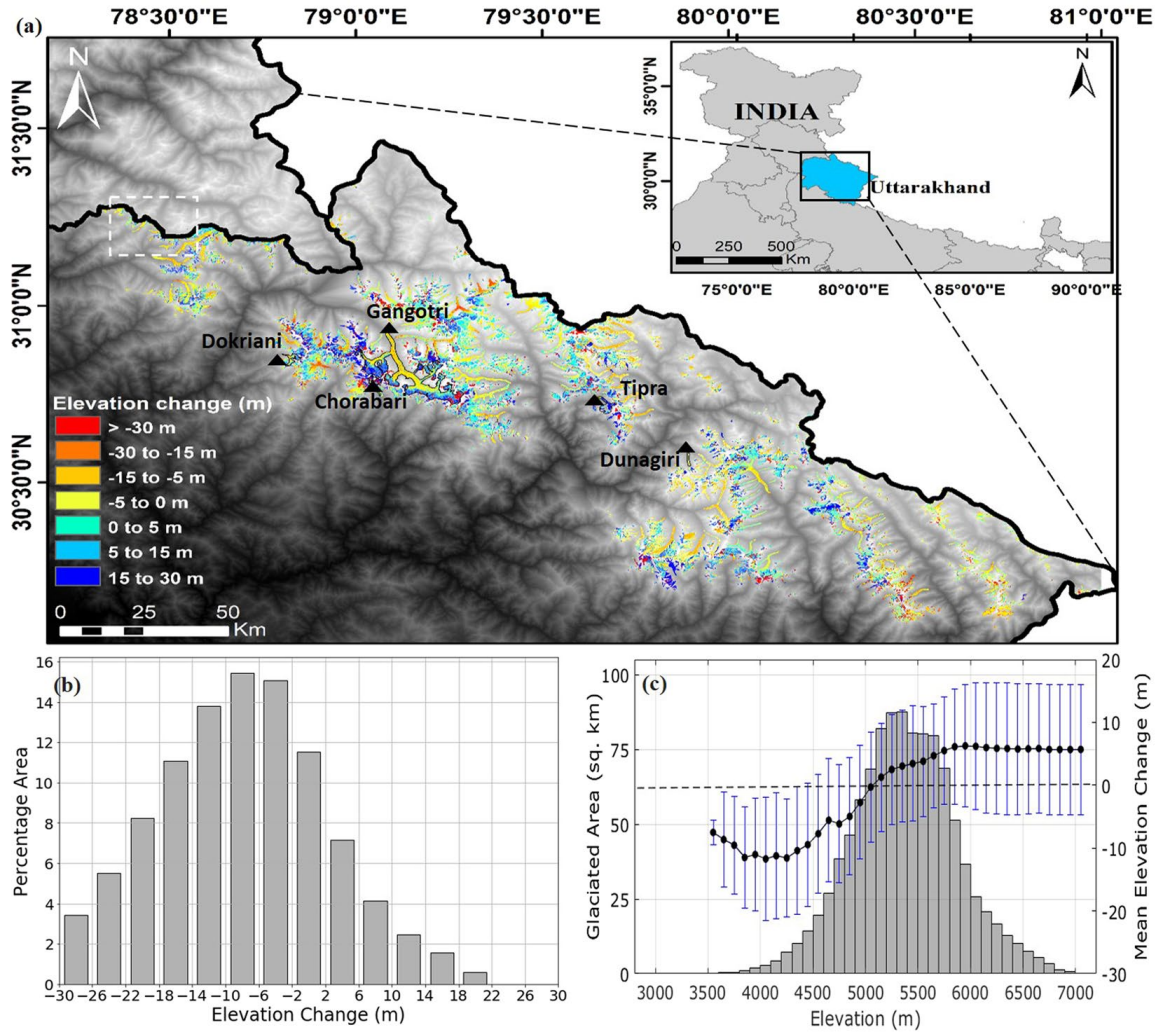
.

## References

[1] Bhushan S, Syed TH, Kulkarni AV, Gantayat P, Agarwal V (2017). Quantifying changes in the Gangotri glacier of central Himalaya: Evidence for increasing mass loss and decreasing velocity. IEEE J. Sel. Top. Appl. Earth Obs. Remote Sens.

