# Supplementary material for: Spatial distribution of decadal ice-thickness change and glacier stored water loss in the Upper Ganga basin, India during 2000–2014
Source: Sci Rep. 2019 Nov 13;9:16730. doi: 10.1038/s41598-019-53055-y (PMC6853911; doi:10.1038/s41598-019-53055-y)
Supplement: Supplementary file 1 — Spatial distribution of decadal ice-thickness change and glacier stored water loss in the Upper Ganga basin, India during 2000-2014 [file 41598_2019_53055_MOESM1_ESM.pdf]

SUPPLEMENTARY MATERIAL

Spatial distribution of decadal ice-thickness change and glacier stored water loss in the Upper Ganga basin, India during 2000-2014

Debmita Bandyopadhyay[1], Gulab Singh[1*], Anil V. Kulkarni[2]
[1]Center of Studies in Resources Engineering, Indian Institute of Technology Bombay, India
[2] Divecha Centre for Climate Change, Indian Institute of Science, Bangalore, India
*Corresponding author Email ID: gulab.singh@iitb.ac.in

Supplementary information includes:

1. DEM bias correction
   1) DEM co-registration
   2) Penetration bias correction (with Supplementary Table S1)
2. Surface elevation change and mass change estimation
3. Uncertainty estimate with supplementary Fig.S1
4. Accumulation Area Ratio (AAR) for 2000 and 2014 (Supplementary Table S2)
5. Catchment-wise elevation change (Supplementary Fig. S2)
6. Surface temperature for Uttarakhand region (Supplementary Fig. S3)
7. Detailed spatial variation of elevation change selective in individual glaciers from each catchment (Supplementary Fig. S4(a-n))

1. DEM bias corrections

The two DEMs (SRTM-C and TanDEM-X) have different frequency, time of acquisition and processing steps are also different which lead to different slope variations. Thus there are certain bias corrections that are required to be performed in order to compare the two dataset for further analysis.

The precision of the DEMs that were generated for Bhagirathi catchment were calculated using the formula used by Deo *et al.* [1] which is as follows:

$$\sqrt{\frac{1-\Upsilon^2}{2\Upsilon^2}\frac{\lambda R\ sin\theta}{4\pi B_{perp}}} \qquad \text{(Equation 1)}$$

where Υ is the coherence of the image, λ is the wavelength of the sensor, θ is the incidence angle and B is the perpendicular baseline.

1) DEM coregistration

Co-registering two DEMs is necessary to avoid biased or erroneous estimation of change in elevation. To reduce the horizontal shift in the two DEMs, first they are brought to the same projection and resampled subsequently. Main principle of co-registration involves minimizing the standard deviation of the elevation difference on the glacier free terrains. This methodology has been well tested for the Himalayan terrain by Berthier *et al.*[2]. The resultant image of co-registration was obtained using the established relationship [3] of elevation difference, slope and aspect, which is given as:

$$dh = a.cos(b - \varphi).tan(\alpha) + \overline{dh} \qquad \text{(Equation 2)}$$

where dh is the individual elevation difference, a is the magnitude of horizontal shift, b is the direction of shift vector, α is the terrain slope, φ is the terrain aspect and $\overline{dh}$ is the overall elevation bias.

2) Penetration bias and seasonality correction

C-band and X- band have differential penetration capabilities on a surface. This property is more enhanced on a dynamic surface like snow which changes on an hourly/daily basis. SRTM- X and SRTM-C dataset over the selected area is considered for penetration correction. Since both datasets are acquired in the same season and time, seasonality correction is not needed in this case. Both glaciated and non-glaciated terrains are considered for the SRTM-X and C-band penetration differences. During this process, glacier outlines from the RGI inventory are utilized to obtain masked region for both the regions (glaciated and non-glaciated). However, with limited coverage of the SRTM-X in the selected region of study, the penetration bias could not be corroborated for the entire region of interest.
The radar penetration bias in glaciers has been found typical to the region characteristics. Vijay and Braun [4] suggest that the penetration bias has a strong dependency on altitude which can be attributed to the surface characteristics of the glacier like presence of dry firn, snow or ice.

The penetration bias for X- and C-band can be estimated only after the DEM biases are removed. The DEM bias creeps in as both the DEMs have been processed differently (SRTM-X at DLR and SRTM-C at NASA). The mean penetration over the glaciated terrain, after DEM bias removal has been estimated as 3.5 m (area covered is marked with white dashed rectangle in Figure 1a). It should be noted that every region has a unique value of penetration bias. Gardelle *et al.* [5] and Vijay and Braun [6] apply penetration difference calculations from one region to another. For instance, the penetration bias for Karakoram Himalaya was applied on Lahaul-Spiti region [4]. This study highlights that the penetration difference of the two bands (X- and C-) varies from region to region. For comparison, Table S1 shows penetration differences for different regions in the Himalayan terrain itself. This observation is particularly important for elevation change estimation, as directly impacts the mass balance measurements.

Region of glacier	Penetration difference (m)	Reference
Lahaul-Spiti (Himachal Pradesh)	0.84 to 3.64	Vijay and Braun (2016)
Pamir (Jammu-Kashmir)	1.8	Gardelle *et al.* (2013)
Karakoram (East and West)	2.4	Kääb *et al.* (2012)
Central Himalaya (Uttarakhand)	3.5	Present study

Table S1 : Average penetration difference of SRTM-X and SRTM-C band over different regions of Indian Himalayan region

The penetration bias of X-band SRTM and X-band of TanDEM-X are assumed to be the same in this study. The dataset (SRTM-C&and-X and TanDEM-X) have been acquired in the same winter

season, hence seasonality corrections have been negated, even though the dataset has been acquired from different sensors and ~~are~~ acquisitions are a decade apart (SRTM in 2000 and TanDEM-X in 2011). This approach has been also adopted in previous studies [4,7,8].

2. Surface elevation and mass change estimation

Differential height calculations for the entire Central Himalayan glaciers was performed using SRTM-X and TanDEM-X DEMs, following the methodology used by Vijay and Braun (2016). This methodology of calculating decadal elevation change forms the prime step in the geodetic method for mass balance estimation. Elevation to volume change is calculated using the glacier boundary used from the modified RGI inventory (6.0) [9]. A threshold of 0.1 km^2 has been applied to the glacier outlines to avoid considering snow patches for current study. Finally for estimating mass change the volume is multiplied with the density[10] of 850 ± 60 kg m^{-3} to cover a wide range of density(ice/snow/firn) in the glaciers.

3. Uncertainty estimate

The measurements taken on surface have an accompanied uncertainty that needs to be accounted for. There are three major sources from which uncertainty arises: (a) elevation change, (b) glacier boundary delineation and (c) mass to volume conversion.

(a) The elevation change using the two-time period DEMs is corrected for using the standard method of estimating the normalized median absolute deviation (NMAD).This method is robust for a distribution with a large number of outliers [11], hence preferred. In this study, the final uncertainty is calculated as:

$$Uncertainity = \frac{\triangle E_h}{\sqrt{N_{eff}}} \qquad \text{(Equation 3)}$$

$$\text{where, } N_{eff} = \frac{P.\ N_{total}}{2d} \qquad \text{(Equation 4)}$$

where, $\triangle E_h$ is the standard deviation of the elevation change on off-glacier points, N_{eff} is given by the total number of observations (N_{total}), the pixel size (P) and the spatial autocorrelation (d). The spatial autocorrelation distance gives an idea about the spatial distribution pattern promoting that the observed pattern of values is a random chance. This means, for a shorter distance of spatial autocorrelation (d), the chance of having a biased elevation change value is higher. We observe that with value of d=1480m, the uncertainty in elevation change values is well within acceptable limits. This approach of accuracy assessment has been used in earlier studies [5,6].

(b) The glacier boundary delineation uncertainty is estimated using the formula used by Braun *et al.*[12] which is as follows:

$$\delta A = \frac{r_{P/A}}{R_{P/A}} \times 0.03 \qquad \text{(Equation 5)}$$

where, δA is the uncertainty owing to the manual delineation of the glacier boundary, $r_{P/A}$ is the ratio of perimeter to area for the glacier delineated and $R_{P/A}$ is a constant value for the alpine catchments with a reported 3% error which is represented by the value, 0.03.

The extent to which the RGI boundaries have been modified is shown in Supplementary Fig. S1. It can be seen in Supplementary Fig. S1a) and c) that there are significant changes in the frontal area. Thus, these changes need to be corrected for, else we might miss out on the elevation change for the frontal part of the glacier, where the maximum elevation change/retreat is observed. Hence, the RGI boundaries had to be modified as per our study period, considering the base year as 2000. Further, the changes are not restricted to frontal areas. As seen in Supplementary Fig.S1b), the RGI 6.0 boundaries shows that the tributary glacier (I) has detached from the main glacier (II), whereas in the year 2000, it was still a part of the main glacier (II). These minute changes when taken into consideration will give a more accurate picture of the ice-thickness changes that have occurred during the observation period, 2000-2014.

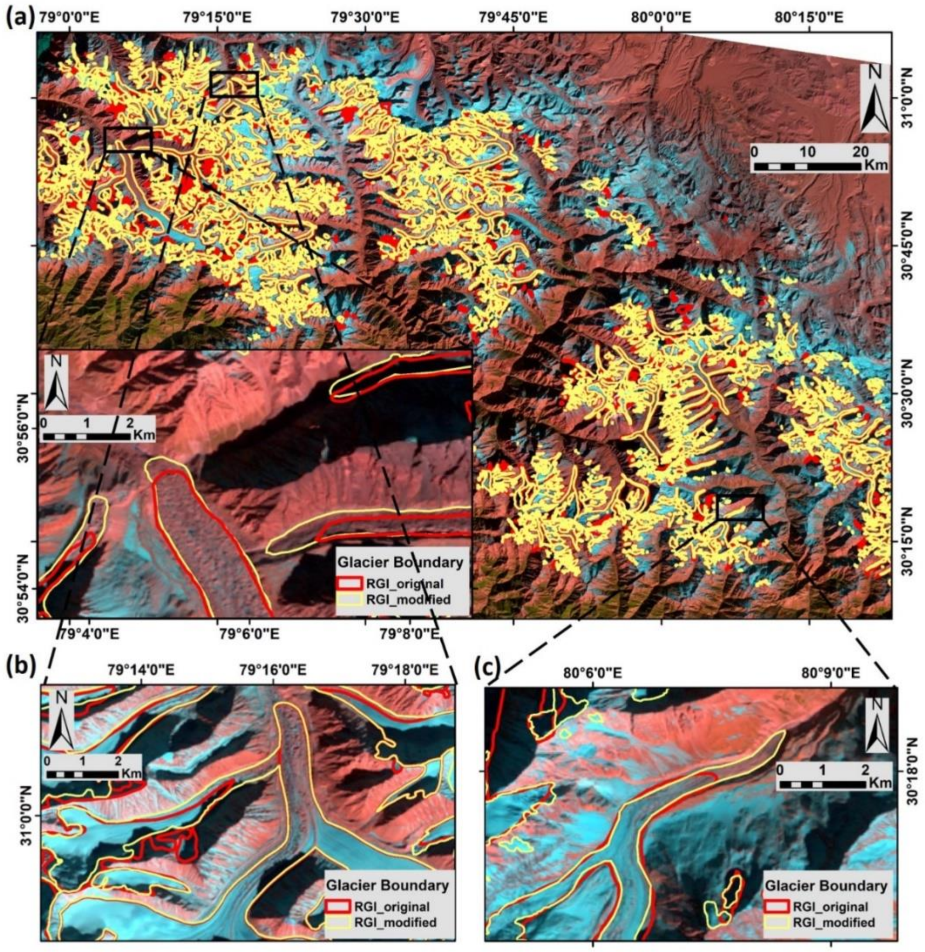

Supplementary **Fig. S1:** RGI boundary (original and manually modified) are shown for a selected region of study in the inset wherein the yellow outline is the RGI boundary modified using the FCC image of Landsat 4/5 of the year 2000(Landsat image courtesy of the U.S. Geological Survey). The red outline signifies the original boundaries provided by the RGI 6.0 inventory, which has been updated constantly from 2001 till 2011.

(c) Uncertainty caused by mass to volume conversion is taken care in the ± 60 kg m^{-3} value considered while using the density value of 850 ± 60 kg m^{-3}. This process of accounting for uncertainty has been reported in Brun et al. [13]

4. Accumulation Area Ratio (AAR) for 2000 and 2014 (Supplementary Table S2)

Catchment name	AAR	
	**Year 2000**	**Year 2014**
Yamunotri	0.596	0.565
Bhagirathi	0.552	0.491
Mandakini	0.306	0.268
Alaknanda	0.389	0.476
Dhauliganga	0.421	0.375
Pindar	0.272	0.112
Goriganga	0.59	0.387
Kali	0.503	0.480

Table S2: AAR for all the catchments to showcase the change from 2000 to 2014.

5. Catchment-wise elevation change (Supplementary Fig. S2)

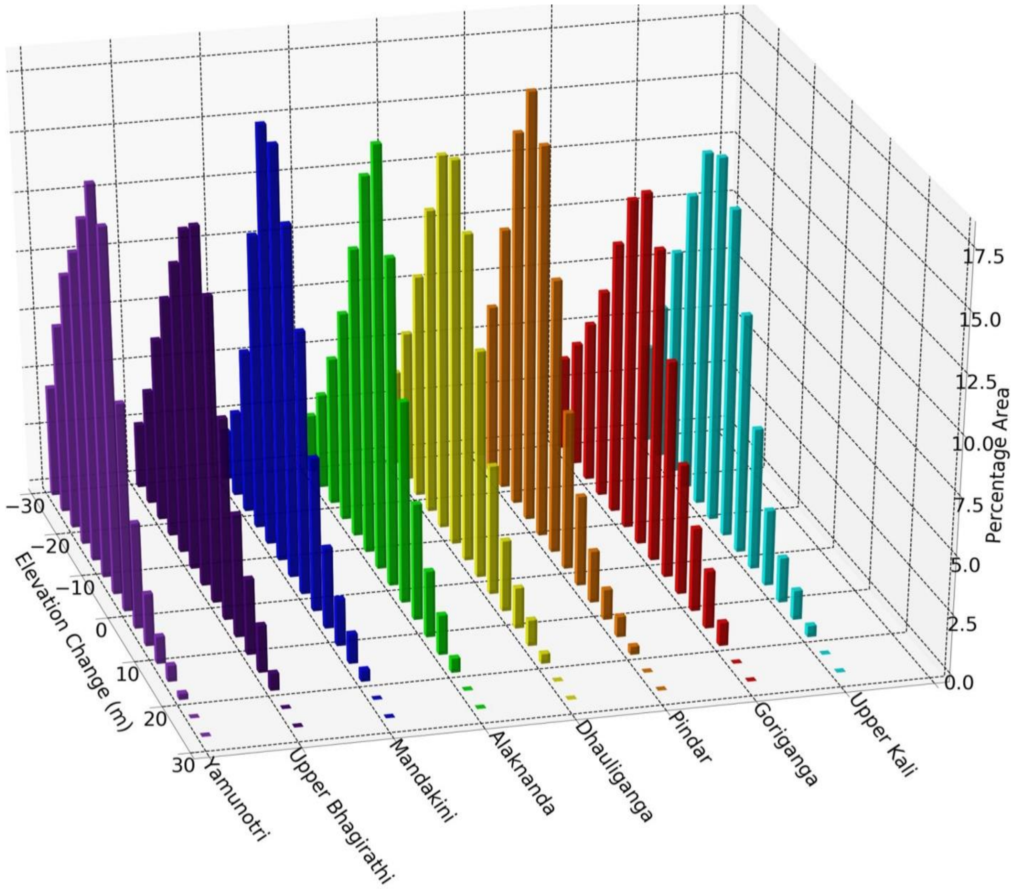

Supplementary **Fig. S2:** Plot of mean elevation change for all eight catchments against the percentage of glacier area each catchment

6. **Surface temperature for Uttarakhand region** (Supplementary Fig. S3)

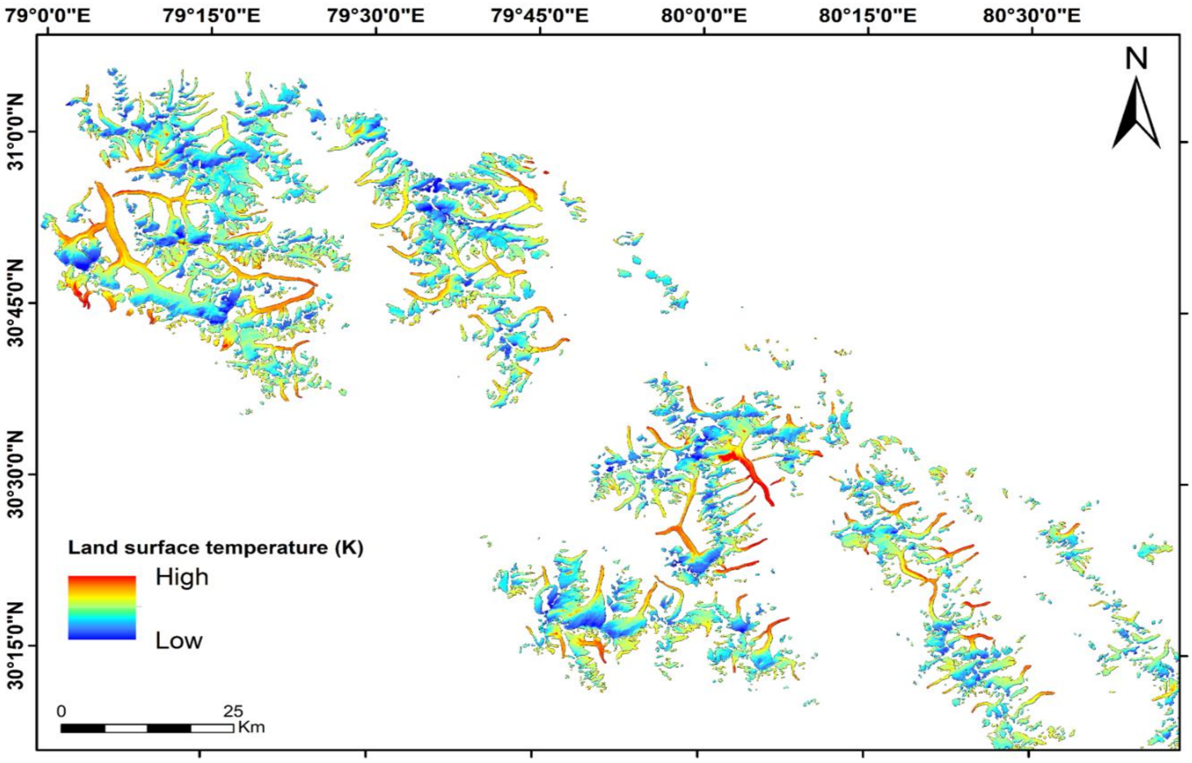

Supplementary **Fig. S3:** Surface temperature (in Kelvin) of glaciers showing that at lower elevation, glaciers have a higher temperature.

**Catchment Name**	**Mean Temperature change (in deg. Celsius) (from ERA5 reanalysis data)**		**Mean temperature change (in deg.Celsius) (from Landsat thermal band)**	
	**2001**	**2014**	**2000**	**2014**
Yamunotri	-	-	-1.92	-0.07
Bhagirathi	4.57	5.32	-2.02	-0.82
Alaknanda	-0.33	0.93	-0.90	-1.85
Mandakini	-	-	0.45	1.01
Dhauliganga	4.78	5.30	0.33	Cloud covered
Pindar	7.15	7.59	1.30	1.75
Goriganga	7.85	8.03	-0.43	0.60
Upper Kali	10.67	11.42	-2.12	0.45

Table S3: Mean monthly temperature for September2000- September 2014, in each catchment is derived Landsat thermal band (30 m spatial resolution) alongwith the temperature changes obtained from climate reanalysis data (ERA5 [14] of 9 km spatial resolution). Note that ERA5 data is available 2001 onwards.

7. **Detailed spatial variation of elevation change in individual glaciers** (Supplementary Fig. S4)

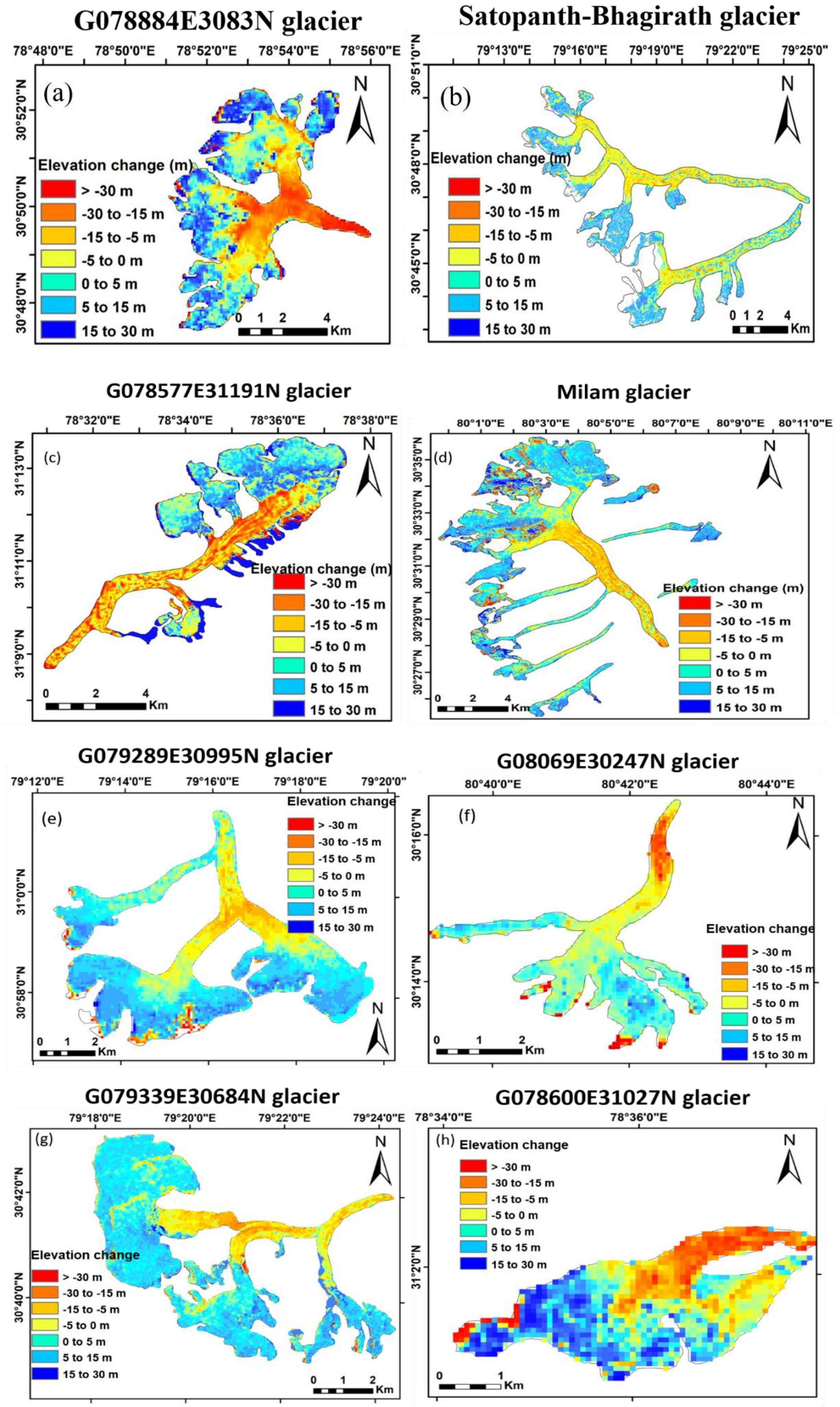

(Supplementary Fig. S4 contd.)

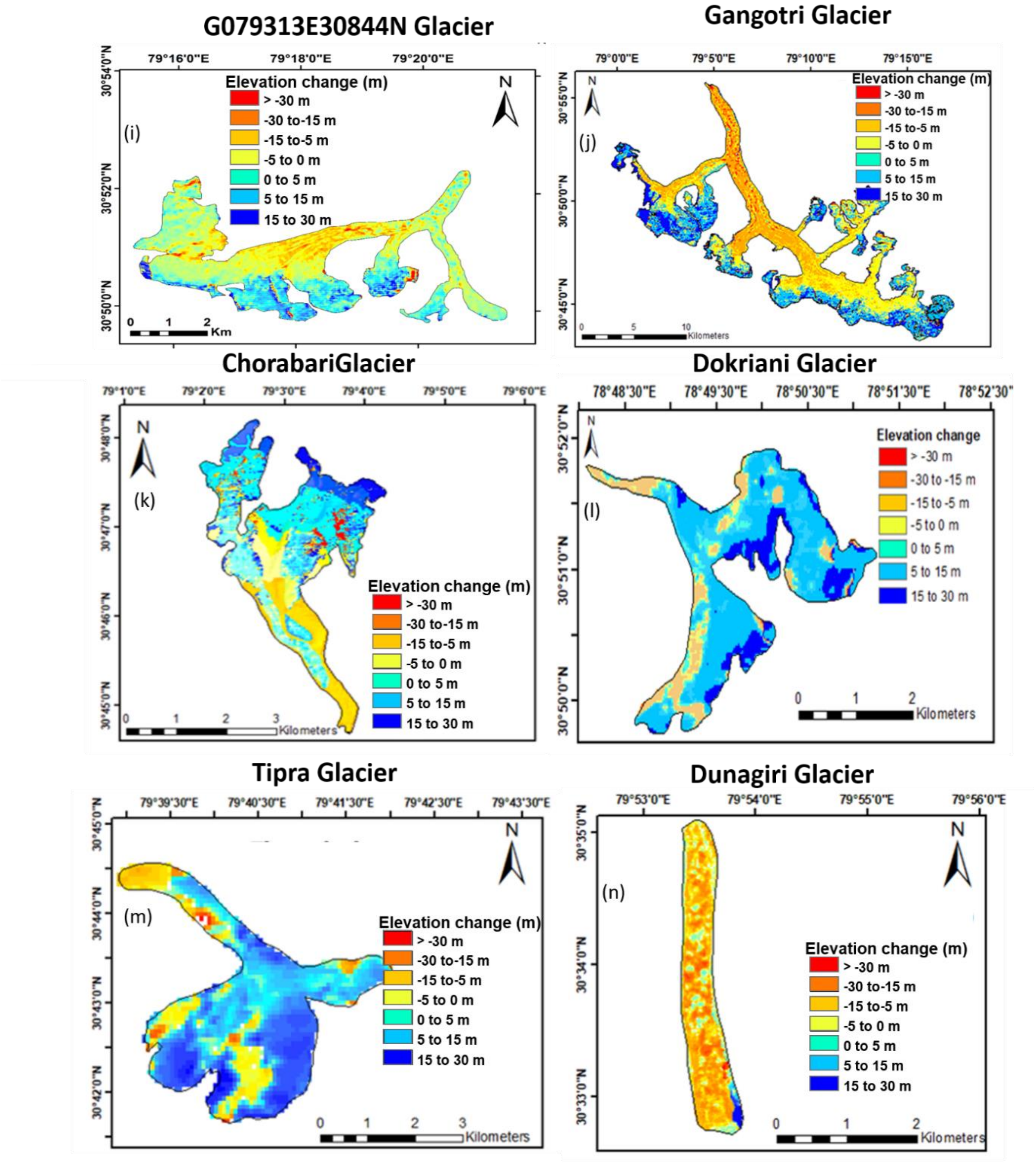

Supplementary **Fig. S2 :** Spatial variability of selective glaciers (a) G078884E3083N glacier from Bhagirathi catchment (b) Satopanth-Bhagirath glacier glacier from Alaknanda catchment (c) G078577E31191N ID glacier from Yamunotri catchment (d) Milam glacier from Goriganga catchment to highlight the variability in elevation change (e) G079289E30995N ID glacier from Bhagirathi catchment (f) G080696E3024N ID glacier from Kali catchment (g) G079339E30684N glacier ID from Alaknanda catchment (h) G078600E31027N glacier ID from Yamunotri catchment (i) G079313E30844N ID glacier from Alaknanda catchment (j) Gangotri glacier from Bhagirathi catchment (k) Chorabari glacier from Mandakini catchment (l) Dokriani glacier from Bhagirathi catchment (m) Tipra glacier from Alaknanda Catchment and (n) Dunagiri glacier from Dhauliganga catchment

References

1. Deo, R., Manickam, S., Rao, Y. S. & Gedam, S. S. Evaluation of interferometric SAR DEMs generated using TanDEM-X data. *International Geoscience and Remote Sensing Symposium (IGARSS)* 2079–2082 (2013). doi:10.1109/IGARSS.2013.6723221

2. Berthier, E. *et al.* Remote sensing estimates of glacier mass balances in the Himachal Pradesh (Western Himalaya , India). *Remote Sensing of Environment* **108,** 327–338 (2007).

3. Nuth, C. & Kaab, A. Co-registration and bias corrections of satellite elevation data sets for quantifying glacier thickness change. *Cryosphere* **5,** 271–290 (2011).

4. Vijay, S. & Braun, M. Elevation change rates of glaciers in the Lahaul-Spiti (Western Himalaya, India) during 2000-2012 and 2012-2013. *Remote Sensing* **8,** 1–16 (2016).

5. Gardelle, J., Berthier, E., Arnaud, Y. & Kääb, A. Region-wide glacier mass balances over the Pamir-Karakoram-Himalaya during 1999–2011. *Cryosphere* **7,** 1263–1286 (2013).

6. Vijay, S. & Braun, M. Early 21st century spatially detailed elevation changes of Jammu and Kashmir glaciers (Karakoram–Himalaya). *Global and Planetary Change* **165,** 137–146 (2018).

7. Rott, H. *et al.* Mass changesof outletglaciers along theNordensjköld Coast, northern Antarctic Peninsula, based on TanDEM-X satellitemeasurements. *Geophysical Research Letters* **41,** 8123–8129 (2014).

8. Rankl, M. & Braun, M. Glacier elevation and mass changes over the central Karakoram region estimated from TanDEM-X and SRTM/X-SAR digital elevation models. *Annals of Glaciology* **57,** 273–281 (2016).

9. RGI Consortium. *Randolph Glacier Inventory – A Dataset of Global Glacier Outlines: Version 6.0.* (2017). doi:https://doi.org/10.7265/N5-RGI-60.

10. Huss, M. Density assumptions for converting geodetic glacier volume change to mass change. *The Cryosphere* **7,** 877–887 (2013).

11. Höhle, J. & Höhle, M. Accuracy assessment of digital elevation models by means of robust statistical methods. *ISPRS Journal of Photogrammetry and Remote Sensing* **64,** 398–406 (2009).

12. Braun, A. M. H., Malz, P., Sommer, C. & Barahona, D. F. Constraining glacier elevation and mass changes in South America. *Nature Climate Change letters* **9,** 130–136 (2019).

13. Brun, F., Berthier, E., Wagnon, P., Kääb, A. & Treichler, D. A spatially resolved estimate of High Mountain Asia glacier mass balances from 2000 to 2016. *Nature Geoscience* **10,** 668–673 (2017).

14. Copernicus Climate Change Service (C3S):C3S ERA5-Land reanalysis. *Copernicus Climate Change Service* (2019). Available at: https://cds.climate.copernicus.eu/cdsapp/home. (Accessed: 29th September 2019)
